# Treatment of Pneumonia at Bellevue

**Published:** 1882-04

**Authors:** 


### THE TREATMENT OF PNEUMONIA AT BELLEVUE.

The motive of the general treatment of pneumonia at Bellevue Hospital is to sustain the powers and stimulate the functions of the patient till the comparately brief and self-limited disease shall have spent itself.

The pulse is taken, rather than the temperature, as the gauge which best indicates the capacity for resistence, and an increase in its rapidity and diminution in its force are understood as a call for stimulants. The forms of stimulation used are to some extent subject to differences of opinion on the part of the visiting physicians, but all are agreed as to the value of wkisky, and there is almost as much unanimity in their regard for the carbonate of ammonium. Digitalis is much used, but it is objected to by some, partly because experience seems to indicate that in some cases, when the crisis of the disease has passed, patients are left, after its use, in a condition less favorable for recovery, and partly from the theoretical consideration that this drug is not general enough it its action. Camphor has been employed by some as a diffusable stimulant.

The general treatment of pneumonia is, then, by simple stimulation. In special conditions, however, more is done. When the patient is first seen, if he is suffering from considerable pain, a few doses of morphia are recommended. If the disease is seen at its outset, and if the outset is violent in character, one at least of the leading physicians on the visiting staff believes in the good effect of a few doses of aconite, but its use is not general in the hospital. The spirit of Mindererus, sweet spirit of nitre, calomel, and Dover's powder, are used by some in the first stage of the disease. Quinine is occasionally called to bring down the temperature when it rises to a serious height. One of the visiting physicians makes a special point of the importance of watching the kidneys and seeing that they perform their duty well.

The appearance of œdema of the lungs finds all agreed upon the necessity of pushing the stimulants. But beyond this there are some differences of practice. They would be included in the use of dry cups, the hot pack, oxygen, and, in the few cases which are entirely suitable for it, bleeding.—*Medical Record.*

